# Supplementary figures and images for: ExprTarget: An Integrative Approach to Predicting Human MicroRNA Targets
Source: PLoS One. 2010 Oct 21;5(10):e13534. doi: 10.1371/journal.pone.0013534 (PMC2958831; doi:10.1371/journal.pone.0013534)

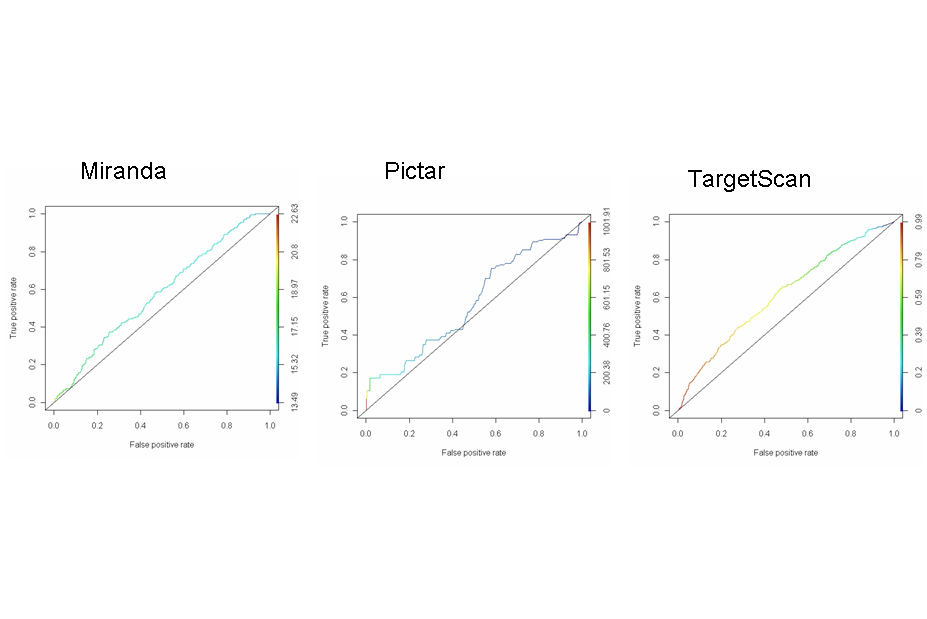

Supplement: Figure S1 — Individual performance of foundational prediction algorithms using (TarBase + LCL) as gold standard. The three prediction algorithms were evaluated using ROC curve analysis, using (TarBase + LCL) as benchmark. (2.30 MB TIF) [file pone.0013534.s001.tif]

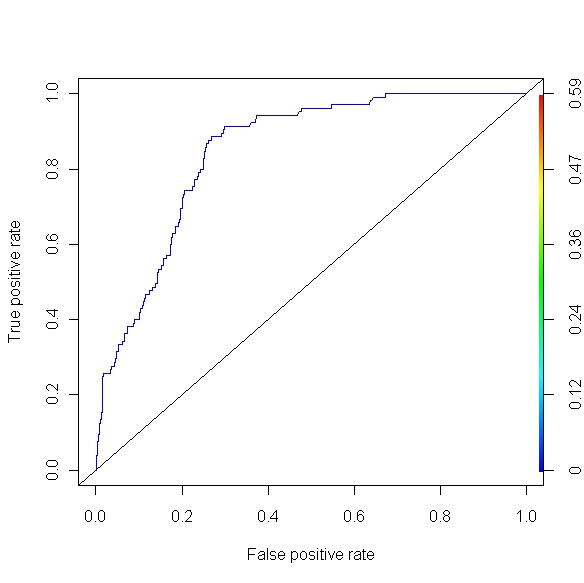

Supplement: Figure S2 — When the subset of TarBase that excludes the high-throughput assays is used as gold standard, the improvement in predictive performance for ExprTarget relative to the individual methods continues to hold robustly. (1.02 MB TIF) [file pone.0013534.s002.tif]

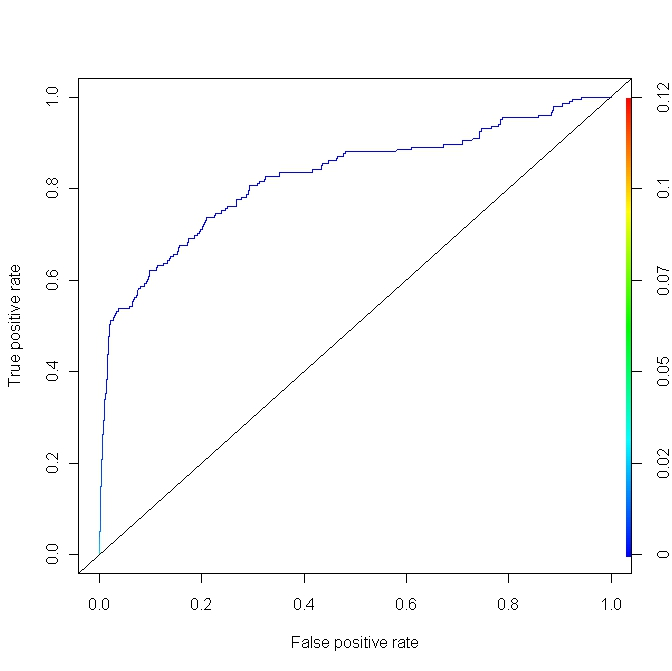

Supplement: Figure S3 — The use of a score from the expression data that is gene-based rather than target site-based (e.g., the score is defined as the minimum of all p values for miRNA correlations with the gene) shows that the incorporation of the individual algorithms improves predictive performance. (1.35 MB TIF) [file pone.0013534.s003.tif]
